# Local and national stakeholders’ perceptions towards implementing and scaling up HIV self-testing and secondary distribution of HIV self-testing by Option B+ patients as an assisted partner service strategy to reach men in Haiti

**DOI:** 10.1371/journal.pone.0233606

**Published:** 2020-05-22

**Authors:** Donaldson F. Conserve, Jacob Michel, Joseph Emmanuel Adrien Demes, Jean Marcxime Chéry, Jean-Gabriel Balan, Augustine Talumba Choko, Kesner François, Nancy Puttkammer

**Affiliations:** 1 Department of Health Promotion, Education and Behavior, Arnold School of Public Health, University of South Carolina, Columbia, South Carolina, United States of America; 2 Family Health International, Port-au-Prince, Haiti; 3 Université de l’État d’Haiti, Port-au-Prince, Port-au-Prince, Haïti; 4 Centre Haïtien pour le Renforcement du Système de Santé (CHARESS), Port-au-Prince, Haïti; 5 Malawi-Liverpool Wellcome Trust Clinical Research Programme, Blantyre, Malawi; 6 Programme National de Lutte contre le SIDA (PNLS), Ministère de la Santé Publique et de la Population (MSPP), Port-au-Prince, Haïti; 7 International Training and Education Center for Health (I-TECH), Department of Global Health, University of Washington, Seattle, Washington, United States of America; University of the Witwatersrand, SOUTH AFRICA

## Abstract

HIV self-testing (HIVST), which allows people to test in private, is an innovative testing strategy that has been shown to increase HIV testing among men. Delivering HIVST kits to men via women is one promising assisted partner service strategy. Little research has been conducted on HIVST secondary distribution to men by women living with HIV (WLWH) in the Caribbean and other settings. The purpose of this study was to assess the perspectives of WLWH, their male partners, and healthcare professionals on the perceived advantages and disadvantages of HIVST, and recommendations for implementing HIVST in Haiti, with a focus on secondary distribution of HIVST to men by WLWH. Sixteen key informant interviews and nine focus groups with 44 healthcare workers, 31 Option B+ clients, and 13 men were carried out in Haiti. Key informants were representatives of the Ministry of Health and of a non-governmental agency involved in HIV partner services. Focus group members included program leads and staff members from the HIV care and treatment program, the Option B+ program, the community health service program, and the HIV counseling and testing services from 2 hospitals. Perceived HIVST advantage included an increase in the number of people who would learn their HIV status and start treatment. The perceived disadvantages were lack of support to ensure self-testers initiate treatment, uncertainty about male partner’s reaction, risk of violence towards women delivering HIVST kits after receiving an HIVST kit from a woman, and the inability of women to counsel a man in case his self-test result is positive. Recommendations for integrating HIVST and secondary distribution of HIVST by WLWH included coupling HIVST distribution with public information, education, and communication through media and social marketing, relying on community health workers to mediate use of HIVST and ensure linkage to care, piloting HIVST programs on a small scale. HIVST is an appropriate and feasible strategy HIV prevention for men and women; however, more research is needed on how best to implement different strategies for this approach in the Caribbean.

## Introduction

HIV prevalence in Haiti has remained relatively stable for the last 10 years at 2.0% [1], among the highest HIV prevalence levels in the Caribbean region. Achievement of the Joint United Nations Programme on HIV/AIDS (UNAIDS) 95-95-95 targets by 2030 hinges upon the first 95 –that 95% of all people living with HIV/AIDS (PLWH) learn their status [2]. Haiti has recorded significant progress in increasing access to HIV testing services within the last decade. HIV testing increased from 5% to 20% among men and 8% to 28% among women between 2005–17 [1]. In 2017, 60% of women reported to have ever tested compared to 41% of men [1]. The persistently high levels of the adult population who have never tested for HIV and the disparity between men and women are concerning for HIV testing and initiation of antiretroviral therapy (ART) [3]. Evidence from a nationally representative sample of heterosexual men demonstrated that condomless sex is high [4] and that HIV testing is associated with less condomless sex [5], indicating the importance of promoting HIV testing among men.

In order to increase HIV testing rates and facilitate early access to treatment, the Haitian Ministry of Health, Ministère de la Santé Publique et de la Population (MSPP), has prioritized finding novel approaches to expanding HIV testing and linkage to care, especially among men. HIV testing among women is a well-established part of antenatal care services (ANC); as of 2016 more than 90% of pregnant and post-partum women were tested for HIV as part of efforts for prevention of mother-to-child transmission of HIV (PMTCT) [6]. In 2012, the MSPP expanded access to lifelong HIV antiretroviral therapy (ART) for all pregnant and postpartum women, regardless of level of HIV disease progression, via the Option B+ program and in July 2016 the MSPP extended universal treatment for HIV to all persons living with HIV (PLWH). Retention on ART at 12 months after starting treatment is estimated at 73.1%, short of the level required for viral suppression [6], and there is evidence that women starting ART during the perinatal period are at elevated risk of loss-to-follow up [3, 7]. Lack of couples HIV testing and disclosure of HIV status between partners is a frequently cited reason for the high rates of ART attrition among Option B+ clients [8]. In this context, finding novel strategies to increase HIV testing among men and to support disclosure of status between partners is needed for progress on the 95-95-95 targets [2].

One promising strategy to reach the first 95 target [9, 10], which has been endorsed by the World Health Organization (WHO) and scaled up incrementally in Haiti since 2017, is Assisted Partner Services (APS) [11]. APS is a voluntary public health service whereby healthcare workers work with newly-diagnosed index clients to identify their sexual partners and close contacts, inform them that they may have been exposed to HIV, and link them to HIV testing services [11]. APS increases disclosure of HIV status by index clients and increases uptake of HIV testing among partners, compared to passive referral for HIV testing by index clients [12, 13]. Other strategies to reach men through their female partners include providing written invitation letters to clinic-based testing services, which are hand-delivered by women to their partners, and using community health workers (CHWs) to trace male partners in the community to invite them to attend clinic-based couple testing services. [14–16].

A second innovative strategy with potential to increase HIV testing is use of HIV self-testing (HIVST). HIVST, which allows people to test for HIV in private, has been shown to be acceptable, feasible, and effective in increasing HIV testing, including among men in Sub-Saharan Africa [17–22]. In 2016, WHO recommended HIVST as a complementary HIV testing approach to reach people (i.e. men) who are not accessing current testing services [11] and released guidelines for planning, introducing, and scaling up HIVST [23]. The benefits of HIVST include privacy, increase of access to HIV testing, earlier diagnosis of HIV and confidentiality of results, and reducing queues for facility-based HIV testing [18]. HIVST can also help bypass barriers such as stigma and discrimination that prevent people from accessing facility-based HIV testing [18]. Recent studies conducted in Tanzania found that more than half of men who had never been tested were willing to self-test [24] and that men are interested in self-testing because of the perceived benefits associated with HIVST such as privacy, confidentiality, and time saving [18, 25].

In the context of higher rates of HIV testing among women, secondary distribution of HIVST kits to men by their female partners [19, 26, 27] is recognized as a specific HIVST implementation strategy. This strategy can be applied via newly diagnosed women living with HIV (WLWH) in conjunction with APS or via HIV-negative women, such as antenatal clinic (ANC) clients [28]. Several studies from Uganda, Kenya, and Malawi have also demonstrated that women-delivered HIVST kits to male partners is acceptable, feasible, and effective in increasing HIV testing among men [26–32]. In a RCT conducted in Kenya, women were randomized to an intervention group that received two oral-fluid-based HIVST kits, instructions on how to use them, and encouragement to distribute an HIVST kit to their male partner or to use both kits for testing as a couple. The control group received invitation cards for their partner to seek facility-based HIV testing [19]. Male partner testing was reported to be higher in the intervention group (91%) than the comparison group (52%) [19]. Women in the intervention group were also more likely to report testing as a couple (75%) than those in the control group (33%) [19], indicating the potential of secondary distribution to increase couple’s HIV testing and HIV status disclosure.

Since the release of the WHO guidelines for HIVST in 2016 and the evidence from several studies, a number of implementation science studies of primary and secondary distribution of HIVST have started [23]. Most notable is the Self-Testing Africa (STAR) project, the largest HIVST project being conducted in Africa (Malawi, Zambia, Zimbabwe, Lesotho, Swaziland and South Africa) [23, 33–35]. More recently, facility-based HIVST compared to provider-initiated testing and counseling was reported to increase HIV testing in a cluster-randomized trial in Malawi [36]. While several HIVST studies, including implementation science projects, have been conducted in Africa and other countries [23], our systematic review of HIV testing interventions literature in the Caribbean [37] revealed that only two studies have focused on HIVST in the Caribbean region [38, 39]. Since the publication of this systematic review, results from a recent study in Puerto Rico revealed that the use of HIVST kits to screen sexual partners was acceptable and helped identify partners of positive HIV status among men who have sex with men (MSM) and transgender women, including sex workers, though some participants faced negative partner reactions when broaching the topic of HIVST [40–44].

While these studies have provided preliminary evidence for HIVST in the Caribbean, heterosexual individuals and PLWH were not included. In addition, little is known about the acceptability and feasibility of secondary distribution by WLWH to their male partners and there is no published literature on the perceptions of local and national stakeholders, including community health workers (CHWs), health program leaders, and clients, about the potential of implementing HIVST and secondary distribution of HIVST in the Caribbean region [37]. The perspectives of CHWs are particularly important because of their central role in community outreach for linkage to care, initiation and retention on antiretroviral therapy (ART) for patients diagnosed with HIV in Haiti [45], and because of their involvement in HIVST initiatives in other settings [35, 46]. In Haiti, the Ministry of Health has recently disseminated guidelines for HIVST to be used in community settings with assistance of CHWs, and targeted projects have focused on social network testing with HIVST targeted to MSM and female sex workers [47]; however, there have not yet been initiatives for secondary or social network distribution of HIVST with heterosexual men.

Based on the effectiveness of HIVST [20] and the interest of Haiti’s Ministry of Health to increase HIV testing among men in Haiti through APS, we conducted formative research with local and national stakeholders to assess perceptions towards integrating HIVST, including via secondary distribution by WLWH to heterosexual men, in Haiti. We also elicited their recommendations for how to implement both primary HIVST distribution and secondary distribution of HIVST for male partners of Option B+ clients in the country. This article builds on previous quantitative research assessing factors associated with HIV testing among men [48] and HIV status disclosure to sexual partners among adult men and WLWH in Haiti [49]. The findings will help to inform future implementation and scaling up of HIVST to increase HIV testing among heterosexual men in Haiti.

## Method

### Sampling

Key informant interviews (n = 16) and focus groups (n = 9) were conducted in 2017 in Haiti. Purposive sampling was used to recruit key informants, including representatives of the MSPP at national, regional, and local levels, a non-governmental agency involved in HIV partner services, and hospital leaders in Cap-Haitien and Port-au-Prince by phone calls and e-mails. Similarly, focus group participants were recruited using purposive sampling in collaboration with the medical director at each hospital. The participants for the focus groups were healthcare workers (n = 44), Option B+ clients (women) (n = 31), and male partners (n = 13) of pregnant or post-partum women living with or without HIV receiving services at two Departmental hospitals in Port-au-Prince and Cap-Haitien. Many of the men recruited were partners of Option B+ clients, who knew their female partners’ HIV status. In this case, Option B+ patients were those who were diagnosed with HIV during ANC or during breastfeeding and who enrolled in Option B+ within the 12 months preceding the study. HCWs included program leads and staff members from the HIV care and treatment program, the Option B+ program, the community health service program, and the HIV counseling and testing service. The focus groups consisted of participants aged 30 years and older ranging from 9–12 members per focus group. The study protocol was reviewed and approved by the University of Washington Human Subjects Division (STUDY00002145) and the Haiti Ministry of Health National Bioethics Committee (Ref: 1718–66). Participants provided written informed consent prior to each interview and received an incentive payment of $15 to offset time and transportation costs associated with their participation.

### Data collection and analysis

The consolidated criteria for reporting qualitative research (COREQ) checklist was used to ensure the reporting of the study procedures and findings are consistent with the guideline (see Additional file 1) [50]. The interviews followed a semi-structured topic guide which covered themes of HIV testing, disclosure of HIV status to sexual partners, and strategies for partner notification, including HIVST. The topic guide had a particular focus on strategies for HIV testing of male partners of women who are diagnosed with HIV during pregnancy or during breastfeeding. The interviews were conducted by authors JEAD and JMC. Authors NHP and DFC observed the first focus group, and NHP observed the first 4 key informant interviews. Both NHP and DFC helped coach JEAD and JMC after the sessions based on the discussion. All authors are trained as medical professionals, social scientists, or public health professionals, and have taken part and published peer-reviewed primary literature in the health sciences. Participants in patient focus group participants were not known to the investigators prior to the study. However, some of the participants in the HCWs focus groups and key informant interviews were known to some of the study team members prior to the study. All participants had the opportunity to ask questions and express concerns during the consent-signing process. The motivations and background of the study team members were made clear to the participants in the consent form as well as the interview guides. The interviews were conducted at a convenient location for the key informants while the focus groups were held at the two hospitals. The purpose of the interviews and focus groups was to elicit opinions from a range of stakeholders about potential strategies for increasing the uptake of HIV testing among male partners. Before beginning the HIVST section of the interview, the interviewer provided key informants, HCWs, Option B+ clients, and men with information about HIVST, information about the research that has been conducted on HIVST in other countries, and showed clips from a short HIVST video, developed by the WHO. The video communicated the need for HIVST and showed an example of both oral and blood-based HIVST kits. After providing information on HIVST, the interviewer continued with the following questions: *What do you think about secondary distribution of HIVST to reach men in Haiti*? *How do you see the implementation of such a strategy in Haiti*? *How to manage cases of men with positive self-test results*? *How do you think a man will respond to a partner who offers him an HIVST kit*? *What do you think of their preference for the delivery of the test kit*: *that women deliver the kits or that men receive a sheet to get the test kit of the clinic (where they can ask additional questions*, *or even use the kit there in front of a counselor)*? *Do you think that HIVST would work in Haiti for men*? *Why or why not*? *What obstacles will men have to do HIVST in Haiti and recommendations*? Interviews were conducted by an experienced qualitative researcher in French and Haitian Kreyòl, and were transcribed into French, and then translated into English. The interviews were audio-taped with permission from participants and lasted 60–90 minutes. The sample of interviews and focus groups was pre-determined and limited by available budget and human resources.

Data saturation was achieved via iterative analysis and coding of transcripts. To ensure reliability and validity, the primary author (DFC) and two of the researchers (JEAD and NHP) independently reviewed the transcripts, focusing specifically on the HIVST section in the transcripts [51]. The research team developed a formal codebook for the deductive codes, based on our previous qualitative research on HIVST [18, 26], with primary and secondary codes definitions and quotes supporting these codes [52]. The main codes from our previous research included reasons for willing to self-test (perceived HIVST advantages), potential concerns with HIVST (perceived HIVST disadvantages), and recommendations for addressing HIVST concerns. A directed content analysis approach was used by applying topical codes that described the stakeholders’ perceptions and recommendations related to HIVST and secondary distribution of HIVST in Haiti [53]. A systematic classification process was used to code and identify emerging themes from the transcripts [53].

## Results

Overall, stakeholders had mixed responses about implementing HIVST as a potential HIV testing strategy in Haiti to reach men. There were different views within and across stakeholders groups. and Some of the stakeholders reported that it would be beneficial to have HIVST as an HIV testing strategy while others were not in favor of the strategy due to the lack counseling and resources to support potential self-testers. Most reported that users would prefer the oral versus the blood HIVST kit: “*The oral test would be more easily acceptable and usable by everyone than blood*.” Among the stakeholders who were not in favor of HIVST, they preferred testing at an institution: “*I am for the person to go to test in an institution*, *we would avoid more damage*, *because he/she will be able to find professionals available for follow up*.” Similarly, several stakeholders had concerns about secondary distribution of HIVST to men by WLWH due to the potential negative reactions and physical abuse by male partners in case their self-test results are positive. To address the low awareness of HIVST since it was not yet available in the country during this study, a few stakeholders recommended that campaigns should be developed to raise awareness about HIVST before piloting different strategies to determine the delivery approach that works the best for each group. Instead of women-delivered HIVST kits, stakeholders recommended that CHWs deliver the HIVST kits to male partners to provide the necessary follow-up support and prevent potential harm to the woman. Stakeholders also recommended working with the formal health sector to prevent non-disclosure of HIVST results and designating a separate section in the hospital for HIVST.

### Perceived HIV self-testing advantages & disadvantages

Though most stakeholders were in favor of HIVST, others reported several perceived disadvantages about HIVST. The perceived benefits mentioned for HIVST were related to its potential to increase the number of people who *learn their HIV status*, allow them *to test in private* and *start treatment* as demonstrated in the following two quotes:

As I said it's not a bad thing, maybe already many people would be interested in practicing self-screening, now they have time to self manage before having any discussion with anyone… It would increase the number of people to get tested and knows their status and to have more people to be able to follow the treatment. (Departmental Health Directorate Representative, Cap Haitien)

I see it's a good method, in case the house is full of people, we could find a remote place to do it even when it's not at home to prevent other people from being suspicious or aware and even publishing you on social media. (Male participant, Port-au-Prince)

The perceived disadvantages for HIVST focused on the potential negative reactions a self-tester may have in case of a positive result. Most stakeholders mentioned *lack of counseling*, *potential transmission to others*, *suicidal ideation*, *HIV nondisclosure due to stigma*, and the *risk of losing people after testing* as disadvantages for HIVST.

I think that the person can become aggressive if he/she sees himself/herself as positive and can infect many other people, especially she is not going to have anybody to talk to her, to moralize her, to educate her, raise awareness about treatment or counseling. (HCW, Port-au-Prince).

The problem is that the person would tend to keep the result for her in case it is positive, personally I would do the same. (Male focus group participant, Port-au-Prince)

The other case is if the man actually does it and finds himself positive, he can say that he will end his life or that he will spread the disease. He will not find psychologists to counsel him and many things could happen. (Option B+ client, Port-au-Prince)

Another HCW described the experience of working with patients who were diagnosed with HIV and did not want to seek care in fear of being seeing at the clinic. The participant continued to describe how self-testers may also choose not to seek care and that even people who are working in HIV programs have reported that they may react similarly due to the stigma associated with HIV:

There are patients who are tested and they say they do not intend to continue coming to the site for follow-up to prevent someone from seeing them and they tell you that they prefer to be killed by a bullet instead of someone being aware that they are sick with AIDS. So even when the person who will have to do the self-test might not intend to distribute the virus, however, he would have preferred to stay at home instead of disclosing his status… I have often heard even from trained people working in the [HIV] program that if they were to become infected by someone, they would kill that person and then they would distribute the virus. So it's not a question of education but rather of stigmatization. (HCW, Cap Haitien)

#### Perceptions towards HIVST secondary distribution

*Secondary distribution preference over invitation to test at clinic*. When participants were asked about their perceptions towards secondary distribution of HIVST kits to men by a female partner (either HIV-positive or negative), there were a mixed range of responses, with a few Option B+ clients and male partners participants reporting that this strategy would be preferred by some men as demonstrated in the quotes below. One woman even asked if the HIVST kit was available for her to bring to her partner.

I will not have any problem with my husband and I think he would have preferred that than to come to the hospital because when I talk to him about coming to take the test, he still hesitates and I think it's because that he is stressed. (Option B+ client, Cap Haitien)

Do you have the present test with you to give me to bring to my husband and then how can we do it? (Option B+ client, Cap Haitien)

In my opinion, it would be better if it were so. The person who would only need to go to a health center to take the medication in case it would be positive in addition to the whole family would have the opportunity to do the test and it would stay between them. (Male participant, Cap Haitien)

In contrast, several HCWs, key informants, and Option B+ clients were concerned about how the male partner may react upon receiving the kit and in case his self-test result is positive. They reported the following challenges and perceived disadvantages: *uncertainty about male partner’s reaction*, *the risk for male partner to hit and abandon women*, and whether women will be able *to counsel their partner* in case of a negative reaction.

*Uncertainty of men’s reaction*. Option B+ clients, HCWs, and program directors reported that it might be challenging for a woman to ask her partner to self-test. In addition, they mentioned that the uncertainty of how a man may react after receiving the HIVST kit from their partner is a concern that is not limited to men in Haiti:

So I imagine that it is not easy for them to ask their partner to do the test even at home, besides we will not know what the reaction of the man is because the person who is tested positive before would be blamed by the other. (Healthcare worker, Cap Haitien)

The woman would have a lot of trouble getting the test to her partner. (Option B+ client, Port-au-Prince)

Yes it is the right concern because the man could be in a depressing situation and he can make a reactive reaction of violence … But the man is the same everywhere, that is to say one can have the same reactions in France, in Africa, in the United States […] everything depends on the context in which the individual evolves. (Healthcare program director, Cap Haitien)

*Risk of violence towards women and abandonment*. Related to the uncertainty of men’s reaction, Option B+ clients, HCWs, and directors reported that the female partner may be at risk of being physically abused and abandoned if the man’s self-test result is positive. These risks are higher for WLWH whose male partners are not aware of their own and their partner’s positive HIV status. These concerns were supported by the experience the HCWs had with other men, including a policeman, who tested positive and had a negative reaction:

In case it is the woman who will have to take the test [to the man] and is already positive, the partner could ask her to do it first, and in case this will confirm her positivity, she could be brutalized and even abandoned. (Option B+ HCW, Port-au-Prince)

There are men in the counseling room who say after receiving their result ''Oh well it's positive, I'll deal with her '' so we do not know what he's going to do to the other being under shock. Once it was the case of a policeman who after receiving his test and was positive believed that it was the woman who transmitted the virus to him and threatened to beat her when he arrived home. You imagine a policeman carrying a gun who was talking like that”. (Healthcare Unit director, Cap Haitien)

*Inability of women to counsel partner*. In the case the male partner has a negative reaction due to a positive self-test result, a couple of healthcare workers mentioned the fact the woman may not be able to provide the man with any post-test counseling and support as another disadvantage of secondary distribution of HIVST kits:

In addition one has to wonder if the woman would be able to support a negative behavior of her spouse after having made the self-test because that can even lead to losing a life for a test. (HCW, Cap Haitien)

After the woman brings the test to her husband, she should be able to handle the post-test phase, talking about counseling and caring that is the exclusively reserved for a professional [to do]. (HCW, Cap Haitien)

### Recommendations for integration of HIVST and secondary distribution

#### Provide HIVST education and promotion

A number of stakeholders made recommendations on how to implement HIVST as well as secondary distribution of HIVST, especially for WLWH. Stakeholders from different groups reported strongly that broad education about HIVST would be needed to promote and raise awareness about HIVST in order to create “fertile territory” for how HIVST should be used, disposed, and describe the steps to follow in case of a positive self-test result. Awareness raising should not only happen with targeted HIVST users but with the broader public.

I see that it's [HIVST] very good too, but there needs to be a lot of awareness given the people who live in very isolated areas who do not have access to information because they do not have any device TV or radio, they should be informed that there is a new way to do the test. (Male participant, Cap Haitien)

For the self-test there must be a lot of education sessions on the waste of the self-test, how to manage it, especially in case there are children at home […]. I think we have to think about it. (Option B+ client, Port-au-Prince)

There should be some kind of awareness that is through the media, in schools, churches. (HCW, Cap Haitien)

I think it would be good to do a mass awareness for this kind of approach so that they know that this method exists, if the person cannot come to be screened by a third person, he/she can have the possibility of having this method at home. (HIV program coordinator, Port-au-Prince)

#### Pilot different strategies

A couple of directors also recommended piloting different strategies with different groups of individuals for a long period of time in order to provide the evidence needed to inform the future implementation and determine which strategy works for each person instead of offering one option.

I would advise that instead when we want to go to the programs we start with small acceptable strategies and we try to take several categories of different people, whether poor, married or unmarried […] and see what types of institution that the person attends etc. and try several small strategies to see what can work better, with that we can come out better with evidence […], but it's not something short, to give a result to a health program, the study for example must last [long]. (HIV Regional Director, Cap Haitien)

We will have to do a test in a certain community and even if it is sampling by cluster to see what it will give, and after, depending on the result obtained, we will be able to extrapolate, if it happens to bear fruit … So I think this strategy is not bad, but we will have to do it really as a pilot before the extrapolate. (Program coordinator, Port-au-Prince)

#### Engage community health workers and formal health sector

The other main recommendation from different groups of stakeholders was to have CHWs assist with delivering the HIVST kits because they will be able to provide the follow-up counseling that may be needed if the self-test result is positive. In particular, a CHW should be involved when a woman living with HIV is invited to deliver an HIVST kit to her partner in order to prevent the man from harming the woman and support the man with the outcome of the self-test result.

So for HIV-negative women it would be interesting to have the woman go with the kit for the men to be screened, but for the women who are infected, we can do this approach with the field agents, that the agents go to offer the test to the man, knowing that the man does not have enough time to come to the level of the institution, because automatically I am tested positive, the man will ask questions, why she came with this kit so that I can do the test? And at that moment the man will investigate the woman. So I think this self-screening strategy would be better to do it with officers who have counseling training to help that person accept the outcome, the person could get stung but in front of the field officer who could be a support for this person after. (Partner services director, Port-au-Prince).

In addition to assisting self-testers with the necessary follow-up services, some CHWs and Option B+ clients reported that having a CHW or another healthcare provider present during the self-testing process can help prevent non-disclosure of HIVST results. They also mentioned the idea of having a HIVST station within the clinic where a trained person could assist self-testers.

The person who would have to do his self-test should let you know that he/she is going to do it and do it under your eyes to prevent him/her from lying to you about the test result, they even lie to you when they’re giving you their address, their number, their name, so you see giving you their self-test result would be difficult …” (CHW, Port-au-Prince)

For the good side of self-test I think we could reserve a space in the hospital called *Auto Depistage (*Self-Testing) where someone who is trained for this would be there to take care of people who come for the self test …The person come to do his test, then we show him to the person in charge of the section who would have to verify with him the result. (CHW, Port-au-Prince)

I would encourage the man to go to test in a health institution where he would find a multidisciplinary team to take care of him, because by doing the test alone at home and the result would be positive, this could lead to a lot of damage. (Option B+ client, Port-au-Prince)

The recommendations to engage CHWs and have a designated HIVST station in the hospital are potential strategies to ensure that self-testers receive the necessary follow-up services such as counseling, confirmatory testing, and linkage to care, if needed.

## Discussion

This paper highlights the perceptions of stakeholders towards integrating HIVST, including secondary distribution of HIVST, as a strategy to increase HIV testing among men in Haiti. Stakeholders’ views on this topic are both important and timely, since the President’s Emergency Plan for AIDS Relief (PEPFAR)—a key source of external funding for Haiti’s national HIV prevention, testing, care and treatment program—has embraced HIVST as a core programmatic pillar for achieving the 95-95-95 targets [54]. To ensure on-going support from PEPFAR, Haiti and other countries were expected to make rapid progress to scale up HIVST in 2019–20. Similar to other studies [55, 56], our findings revealed that while some stakeholders had favorable views towards HIVST as a potential strategy to reach men others were skeptical. Reasons for stakeholders’ concerns about HIVST and secondary distribution were related to the lack of follow-up services for self-testers, potential harm for WLWH who may deliver HIVST kits to their male partners, non-disclosure, and linkage to care among self-testers who receive a positive self-test result. In order to address these concerns, some stakeholders recommended several strategies and cited the need to raise awareness about HIVST and conduct pilot studies of different HIVST delivery methods.

Regarding the perceived advantages of HIVST, a few stakeholders, including Option B+ clients and male partners, mentioned that HIVST would allow men to test in private, increase the number of people who learn their HIV status and initiate treatment. Our findings also revealed that Option B+ clients and male partners perceived that secondary distribution of HIVST to men by their female partners would be acceptable and preferred over testing at the clinic for different reasons, including the removal of the stress associated with facility-based testing and the ability to keep the test result private. Most importantly, this study also revealed that a few of the Option B+ patients were willing to deliver an HIVST kit to their partners. These findings are supported by a study in Malawi demonstrating that secondary distribution by WLWH to their male partners was acceptable and feasible [57]. While studies in other low-resource settings have reported similar perceived advantages towards HIVST in general [22], the acceptance of secondary distribution of HIVST by women to their male partners appeared to be higher in other studies than we found in Haiti [58, 59]. One major reason for this difference in acceptability of secondary distribution of HIVST is that our study recruited WLWH while other studies included women who were not living with HIV [58, 59]. In addition, previous trials of secondary distribution of HIVST to male partners included mostly women whose HIV status were unknown or were HIV-negative [27, 29, 60, 61]. Thus, the findings from these trials are not necessarily applicable for WLWH. Additional studies are needed globally to examine the acceptability and feasibility of secondary distribution of HIVST to men by WLWH.

Concerning the perception that HIVST can increase the number of people who test for HIV, this finding is supported by a systematic review and meta-analysis that showed that HIVST doubled the uptake of testing among men [20]. However, linkage to care among self-testers has not been examined in most studies and more data are needed to support our finding that more people will start treatment if they learn their HIV status through self-testing. One study conducted with men in Malawi found that men who self-tested were more likely to attend the clinic for linkage to care or prevention if they received a financial incentive compared to men receiving standard services without an incentive [62]. The likelihood of self-testers not linking to care in case of a positive result was raised as a perceived disadvantage in our study and is supported by evidence from other studies that have demonstrated that self-testers are less likely to link to care without additional interventions such as financial incentive or home initiation of ART [62, 63]. Future research in Haiti and elsewhere should implement strategies to objectively measure HIVST use and linkage to care among self-testers.

Other concerns raised by stakeholders about HIVST and secondary distribution of HIVST by WLWH included, but were not limited to, lack of counseling, suicidal ideation, HIV nondisclosure, the risk for a man to be verbally and physically abusive. These concerns were raised by health program managers, HCW, and Option B+ clients alike. While all the concerns about HIVST in general are consistent with the literature and with concerns raised in Sub-Saharan African settings prior to scale-up of HIVST programs [22], the evidence base on secondary distribution of HIVST by WLWH to their male partners is less robust. In our study in Haiti, all groups of stakeholders recognized that access to HIVST via secondary distribution would be a favorable option for some clients, and several Option B+ clients expressed strong enthusiasm for this option. Therefore, more research is needed to determine how best to implement such a strategy in Haiti and other settings with moderate or low overall HIV prevalence, especially for men who are unaware that their female partners are living with HIV.

In this study, stakeholders recommended several strategies to address the concerns before implementing HIVST in Haiti. These recommendations are broad and can be applied towards implementing both primary and secondary distribution of HIVST, especially for male partners of WLWH. Specifically, stakeholders recommended campaigns to raise awareness and educate people about HIVST, piloting of different strategies, and engaging the CHWs in conjunction with the formal health sector. There is potential for Haiti to broaden its model of using HIVST to increase HIV case detection. Different HIVST campaign strategies and distribution models for HIVST, including self-testing with supervision from formal health sector workers [64], have been piloted and evaluated successfully in RCTs conducted in other countries [19, 65]. Volunteer residents, community HIV care providers, women, and peer educators have been engaged to promote HIVST by distributing flyers and HIVST kits to community residents, male partners, and peers [19, 30, 46, 63, 66–68]. Fishermen living with and without HIV have been recruited and trained to promote HIVST among their peers by distributing leaflets and HIVST kits to other fishermen in their networks in Uganda [17].

In Haiti, men from formal and informal employment sectors, such as public transportation drivers, fishermen, schoolteachers, can also be trained to promote and distribute HIVST kits to their peers and younger men. CHWs already involved in providing community HIV services in Haiti [45] can also be engaged, as recommended by some stakeholders, to promote HIVST and distribute HIVST kits to community residents, including men via either primary distribution or secondary distribution. Other campaign strategies that have been used to promote HIV testing that can be translated to HIVST promote include social entrepreneurship [69, 70], crowdsourcing [71], and hosting free star-studded concerts with concurrent HIV testing and education [72]. In addition, evidence from Kenya, Uganda, and Zambia indicate that HIVST is acceptable and feasible among users of pre-exposure prophylaxis (PrEP) [73, 74]. As the MSPP begins PrEP implementation in Haiti, HIVST could be incorporated in the PrEP program to reduce the number of facility-based visits for HIV testing to confirm PrEP users are HIV-negative before receiving PrEP refills [74, 75]. In summary, stakeholders in Haiti endorsed several recommendations which have a solid base of evidence in other settings, suggesting that the MSPP can draw upon WHO guidelines for planning, introducing, and scaling up HIVST [23] while also seeking culturally and contextually-appropriate implementation strategies for these recommendations.

### Limitations

Though our findings support other similar studies, we only interviewed stakeholders in two regions and therefore the findings may not be representative of stakeholder views in all regions of Haiti. In addition, we only explored opinions towards secondary distribution by WLWHs as opposed to by women in general or male peers. The evidence from the recent male peer delivered HIVST trial indicates that male peer delivered HIVST kits, including by men living with HIV, can reach men without putting their female partners at risk and may be more acceptable to clients [17]. However, these strategies might not support disclosure of HIV status to partners. Future studies HIVST in Haiti should involve stakeholders at different levels and across different regions of the country, to ensure they contribute their expertise for the successful implementation of HIVST in Haiti and other Caribbean countries. Future studies should also explore feasibility and acceptability of additional implementation strategies for primary and secondary distribution of HIVST.

## Conclusion

In summary, this study indicates that a few stakeholders are supportive of implementing HIVST in Haiti. However, there were several areas of important skepticism about the strategy. A prominent concern was due to the lack of follow-up services to assist self-testers to receive proper counseling and enroll on treatment in case of a positive self-test result. In addition, many stakeholders reported concerns about secondary distribution of HIVST by WLWH to their male partners who are unaware their women are living with HIV. Our findings suggest the need for more research in Haiti to determine how to address these concerns such as potential physical and social harms related to HIVST, especially for WLWH who may engage in secondary distribution of HIVST.

## Supporting information

S1 Fig COREQ checklist. (DOCX)
